# Mediating effects of sleep duration on the association between natural menopause and stroke risk among Chinese women

**DOI:** 10.3389/fnins.2022.960497

**Published:** 2022-08-11

**Authors:** Xingyue Liu, Juhua Zhang, Shuzhi Peng, Mengyun Pei, Chunying Dai, Tingting Wang, Peng Zhang

**Affiliations:** ^1^Graduate School, Shanghai University of Medicine and Health Sciences, Shanghai, China; ^2^Graduate School, Shanghai University of Traditional Chinese Medicine, Shanghai, China; ^3^Department of Integrated Traditional Chinese and Western Medicine, Shanghai University of Medicine and Health Sciences Affiliated Zhoupu Hospital, Shanghai, China; ^4^Department of Medicine, Kashgar Vocational and Technical College, Kashgar, China; ^5^School of Nursing and Health Management, Shanghai University of Medicine and Health Sciences, Shanghai, China; ^6^School of Clinical Medicine, Shanghai University of Medicine and Health Sciences, Shanghai, China

**Keywords:** natural menopause, sleep duration, stroke risk, women, mediation

## Abstract

**Background:**

Sleep disturbance is commonly reported by menopausal women. Stroke risk and poor stroke outcomes in women have usually been attributed to menopause. This study aimed to investigate the mediating effect of sleep duration on relationship between menopause and risk of stroke in natural menopause women.

**Materials and methods:**

A cross-sectional study was performed, and participants were recruited through a multistage, stratified, probability proportional to size sampling method in this research. The stroke risk was measured using the risk assessment form for high-risk stroke population. The average sleep duration was calculated by adding up night sleep and afternoon nap duration. Multivariate regression analysis was conducted to identify the association between menopause, sleep duration, and stroke risk. The direct and indirect effects of menopause on stroke risk were analyzed by using the sleep duration in a mediation framework.

**Results:**

Perimenopause, menopause, average sleep duration, and night sleep duration were significantly associated with stroke risk (*P* < 0.001), after adjusting for covariates. Perimenopause and menopause were significantly related to average sleep duration (*P* < 0.001) and night sleep duration (*P* < 0.001). The average sleep duration (ab = 0.016, 95% CI: 0.003, 0.030; ab = −0.048, 95% CI: −0.070, −0.027) partially mediated the relationship between menopause and stroke risk. And night sleep duration (ab = 0.024, 95% CI: 0.009, 0.040; ab = −0.054, 95% CI: −0.077, −0.033) played a major mediating role, in which night sleep duration of ≤5 h mediated the link between both perimenopause (ab = 0.707, 95% CI: 0.392, 1.021) and menopause (ab = −0.787, 95% CI: −1.096, −0.478) and stroke risk; both night sleep duration of >8–9 h (ab = 0.079, 95% CI: 0.010, 0.193) and >9 h (ab = 0.379, 95% CI: 0.086, 0.712) had mediating effects on perimenopause and stroke risk.

**Conclusion:**

A significant relationship between menopause and stroke risk factors among natural menopausal status was found in this study. The average sleep duration, especially night sleep duration, partially mediated the association between menopause and stroke risk, which is a novel insight to the progression of stroke risk in Women. Suitable prevention methods and interventions for sleep in menopausal women may reduce the risk of stroke.

## Introduction

Stroke is the rapid onset of a neurologic impairment caused by an interruption in blood flow to the brain lasting more than 24 h, with high morbidity, disability, mortality, recurrence, and economic impact (Katan and Luft, 2018). It is a sexually dimorphic illness that is a primary cause of death and disability in adults in China (Chauhan et al., 2017; Wu et al., 2019; Yang et al., 2020). Premenopausal women had a far lower incidence of stroke than young males, but the rate of stroke doubles during the menopause transition (Branyan and Sohrabji, 2020; Sandset and Ferretti, 2021). In addition to greater risk, studies on stroke risks are poorer in women than in males (Bushnell et al., 2018; Tanlaka et al., 2020). Even once age is taken into consideration, women account for 60% of stroke-related fatalities (Kaul and Sia, 2021). The involvement of gonadal hormones in stroke etiology, incidence, and prognosis is linked to this sex difference, which correlates to estrogen reduction in women going through menopause (Stewart and Sohrabji, 2020).

Natural menopause, defined as the lack of monthly cycles for at least 12 months, indicating the end of reproductive life due to ovarian failure and a decline in hormone levels (Mishra et al., 2019). The physiologic alterations of menopause are centered on reduced endogenous estrogen production, which is a symptom of reproductive and somatic aging (Allais et al., 2018). Estrogen has beneficial and protective effects on the cardiovascular and cerebral vascular systems; however, as menopause occurs, this estrogenic advantage is lost (Sabbatini and Kararigas, 2020). Menopause was also found to be a risk factor for cardiovascular disease due to estrogen deficiency, particularly in early menopausal women, according to several studies (Bai and Wang, 2020; Su et al., 2021). As a result, menopause is a significant sex-specific risk factor for stroke later in life in women.

Sleep and menopause, which both account for a third of a woman’s life span, are of great concern for the higher risk of stroke and long-term health (Kravitz et al., 2018). Sleep disorder, particularly nighttime awakenings, is one of the most common menopausal symptoms, as well as one of the most reported health problems in peri- and postmenopausal women, causing substantial distress and affecting women’s daily functioning (Beverly et al., 2020; Proserpio et al., 2020). In recent decades, suboptimal sleep duration has been linked to stroke risk factors. Several studies have found a curvilinear relationship between sleep duration and stroke risk; however, there are discrepancies in the literature supporting this association, which might be J-shaped (Akinseye et al., 2016), U-shaped (Fang et al., 2014), or neither (Wang et al., 2022). Sleep duration has long been considered a significant risk factor for stroke.

After menopause, the load of vascular risk factors rises, and one in every five women and one in every six men over the age of 55 will have a stroke incident throughout their rest lifetime (Barker-Collo et al., 2015). The expected rise in the global average age of the female population will increase the occurrence of stroke in women, posing a challenge to our healthcare systems. Consequently, it is critical that researchers take note of this situation by exploring how menopause affects stroke risk.

Despite the fact that menopause has been connected to an increased risk of stroke incidence and all-cause mortality, and sleep duration has been significantly linked to menopause and stroke risk (Muka et al., 2016b), there has been little research into the simultaneous association among menopause, sleep duration, and stroke risk. Napping after lunch is a habitude in Chinese culture, with a prevalence rate of 57.7% (Li et al., 2017). A growing body of research suggests an association between daytime napping and stroke risk (Yamada et al., 2015; Li et al., 2018). The purpose of this study was to examine the associations among natural menopause, sleep duration and stroke risk in Chinese suburban women, and to determine whether the association were mediated by average sleep duration (with a nap), night sleep duration, and afternoon nap.

## Materials and methods

### Participants

This cross-sectional study was undertaken in Shanghai’s Fengxian District, a representative suburban district, from December 2018 to April 2019. Residents aged 35–80 years old who have lived in Fengxian District for more than 6 months were the target group. People who did not reside in the region, with hysterectomy/oophorectomy, and those who refused to participate or sign the informed consent form were excluded. People with difficulty in communication due to serious physical or mental illness were also excluded. This study was approved by the Ethics Review Committee of the Shanghai University of Medicine and Health Sciences, and conducted in an anonymous and volunteer manner. All participants signed a permission form authorizing data collection prior to the study. They were also informed of its purpose and the option to participate or withdraw at any time during the experiment. Participants were divided into premenopausal, perimenopausal, and postmenopausal groups based on their menstrual status, with age interval of 35–40, 40–65, and 42–80 years, respectively.

### Instruments and measurements

#### Self-made questionnaire

Data on the sociodemographic characteristics were obtained using a comprehensive and self-made questionnaire that women answered to identify their age, education, marital status, employment, smoking, height (m), and weight (kg) and menopausal status (defined as absence of menstruation over a period of 12 months). The body mass index (BMI) was computed as weight/height^2^ (kg/m^2^), and the BMI of ≥26 kg/m^2^ was considered to be obviously overweight or obese by the stroke risk assessment form.

#### Stroke risk

The following eight risk factors were assessed using the risk assessment form for the high-risk stroke population developed by the Stroke Prevention and Treatment Engineering Committee of the National Health and Family Planning Commission of China (Longde et al., 2015): (1) Hypertension (a history of high blood pressure of ≥140/90 mmHg reported by the participants or diagnosed by physicians) or use of antihypertensive medicines; (2) Heart disease (diagnosed by physicians), such as atrial fibrillation and/or valvular heart disease; (3) Smoking (consistent or cumulative use of any tobacco product for more than 6 months throughout a lifetime); (4) Dyslipidemia (medically determined); (5) Diabetes (a past diagnosis of diabetes mellitus, or a fasting plasma glucose level of ≥7.0 mmol/L or a random blood glucose level of ≥11.0 mmol/L); (6) Physical inactivity (physical exercise was defined as 30 min of physical activity over three times a week for more than a year, according to the National Physical Fitness Monitoring Program of 2000; physical labor in agriculture was also considered as physical activity; thus, the others were classified as physical inactivity); (7) Apparent overweight or obesity (BMI of ≥26 kg/m^2^); (8) Family history of stroke. The stroke risk factors were graded as follows: high-risk group, with any three or more risk factors from the above eight categories; medium-risk group, suffering from chronic diseases (hypertension, diabetes, and/or heart disease) but with fewer than three risk factors; low-risk group, with fewer than three risk factors but no chronic diseases.

#### Sleep durations

To estimate the average sleep duration, participants were asked through questionnaire about their night sleep duration (the period between going to bed at night and getting up in the morning, excluding awaking time) and afternoon nap duration over the preceding month. The average sleep time was the sum of the two periods.

### Data collection

Permission was sought from the village officials to access their residences for data gathering and for inhabitants to participate. The interviewers were medical students who received a 2-week uniform formal training for conducting the interviews before the formal study. They were accompanied by leaders of the village to visit each household, explaining the purpose and scope of the research, and instructed literate residents on how to fill out the questionnaires, while illiterate individuals were interviewed face-to-face. To verity the consistency, correctness, and integrity of the questionnaire, the data analyst as a quality control person chose 5% of participants to repeat surveys and exclude questionnaires with large disparities.

### Sampling method

**FIGURE 1:**
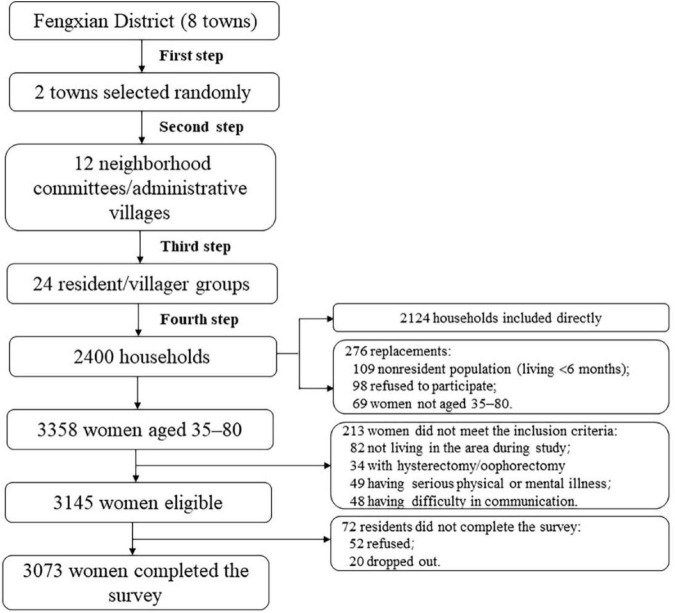
Flowchart of sampling method.

A multistage, stratified, probability proportional to size sampling was adopted to identify participants. In the first stage, two towns were chosen at random out of eight towns in the Fengxian District. In the second stage, six neighborhood committees/administrative villages were selected randomly from each town. Each neighborhood committee/administrative village picked two resident/villager groups consisting of more than 100 households at random in the third step. 100 households were picked at random from each resident/villager group in the fourth step. One to two participants per household were surveyed. The selected households were replaced if they did not meet the inclusion criteria or refused to be surveyed in the field. Residents from the same resident/villager group as the investigation families or from surrounding resident/villager groups were chosen during the replacement method based on the idea of living nearby and having a similar family structure. In the end, 276 households were replaced, for an 11.50% replacement rate (Figure 1).

### Statistical analysis

The data collected was entered and analyzed by the Statistical Package for Social Sciences (SPSS) version 25.0 for Windows. The Kolmogorov–Smirnov test was used to determine the normality of continuous variables. The mean with standard deviation (SD) was used to describe variables that followed a normal distribution, whereas the median or interquartile range (IQR) was used to represent variables that did not conform to a normal distribution. The frequency and percentage were used to express the category variables. The S–N–K test and one-way analysis of variance (ANOVA) were used to evaluate continuous data, while Pearson’s chi-square was used to assess categorical variables.

All analyses were adjusted for, education, marital status, and employment. After adjusting for covariables, we utilized ordinal logistic regression analysis to identify the association between menopause, sleep duration and stroke risk, and general linear regression to explore the relationships between menopause and sleep duration. Mediation analysis was used to investigate the function of the mediating variable (M) in the relationship between the independent variable (X) and the outcome variable (Y). The mediation model was created using Andrew F. Hayes’ PROCESS macro version 3.5. The independent variable was menopause, while the dependent variable was stroke risk. The mediating factors were average sleep length, including night sleep duration, and afternoon nap. Menopause’s total effect (path c) on stroke risk was the sum of the direct and indirect effects. The direct effect (path c′) was the effect of menopause on stroke risk after adjusting for average sleep duration. The indirect effect (path ab) was the mediating impact of the association between menopause and stroke risk. Furthermore, night sleep duration and afternoon naps were examined independently as potential mediators of menopause and stroke risk. We employed the bootstrap method of the PROCESS macro to confirm the statistical significance of the mediating effect. A 95% confidence interval was calculated using 1,000 bias-corrected bootstrap samples to assess the indirect impact (mediation effect). We utilized the 95% CI excluding zero to evaluate if the mediating effects were statistically significant, as advised by Hayes. A *p*-value of <0.05 was considered statistically significant.

## Results

**FIGURE 2:**
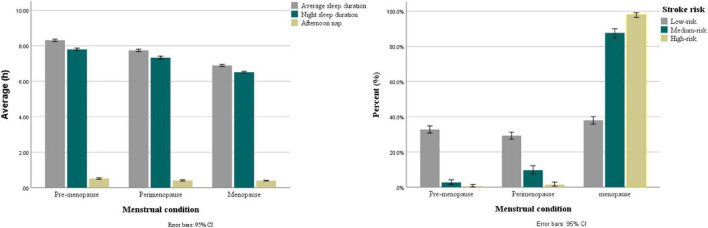
Sleep duration and stroke risk of different menstrual condition.

This study included 3,073 women (1,738 menopause, 656 perimenopause, and 679 pre-menopause), with a mean age of 55.34 ± 11.31 years. Table 1 showed the sociodemographic characteristics and stroke risk factors of the participants. There was no significant difference in the marital status or family history of stroke among the menopause, perimenopause, and pre-menopause groups. The risk of stroke was low (44.0%), medium (31.9%), and high (24.1%) in the menopausal group, while the vast majority are low-risk (93.6%) in the perimenopausal and pre-menopause groups, accounting for 89.8 and 97.2%, respectively, indicating a statistically significant difference between these three groups. The level of average sleep duration and night sleep duration were statistically different in all three groups (*P* < 0.001), with the shortest in the menopausal group (average sleep: 6.90 ± 1.20; night sleep: 6.52 ± 1.09) and the longest in the pre-menopausal group (average sleep: 8.32 ± 0.88; night sleep: 7.80 ± 0.87). whereas the average level of afternoon nap showed no significant difference, as presented in Figure 2.

**TABLE 1:** Sociodemographic characteristics and stroke risk factors.

Variable	Frequency (%)			*P*
	Pre-menopause (*n* = 679)	Perimenopause (*n* = 656)	Menopause (*n* = 1,738)	
Age	(39.93 ± 3.04)	(50.52 ± 4.27)	(63.18 ± 6.74)	<0.001
Education				<0.001
Primary and below	13 (1.52%)	80 (9.33%)	764 (89.15%)	
Middle and high school	151 (11.34%)	326 (24.49%)	854 (64.16%)	
College and above	515 (58.19%)	250 (28.25%)	120 (13.56%)	
Marital status				0.337
With partner	615 (22.24%)	598 (21.63%)	1,552 (56.13%)	
Single/widow/separated	64 (20.80%)	58 (18.83%)	186 (60.39%)	
Employment				<0.001
Stable	499 (40.89%)	325 (26.62%)	397 (32.51%)	
Unstable	180 (9.72%)	331 (17.87%)	1,341 (72.41%)	
Stroke				<0.001
Yes	0 (0.00%)	2 (1.14%)	174 (98.86%)	
No	679 (23.44%)	654 (22.58%)	1,564 (53.99%)	
Hypertension				<0.001
Yes	16 (2.12%)	51 (6.76%)	687 (91.11%)	
No	663 (28.59%)	605 (26.09%)	1,051 (45.32%)	
Dyslipidemia				<0.001
Yes	11 (3.29%)	22 (6.59%)	301 (90.12%)	
No	668 (24.39%)	634 (23.15%)	1,437 (52.46%)	
Diabetes				<0.001
Yes	4 (1.00%)	17 (4.26%)	378 (94.74%)	
No	675 (25.24%)	639 (23.90%)	1,360 (50.86%)	
Atrial fibrillation				<0.001
Yes	3 (1.24%)	10 (4.15%)	228 (94.61%)	
No	676 (23.87%)	646 (22.81%)	1,510 (53.32%)	
Smoking				0.01
Yes	3 (6.82%)	4 (9.09%)	37 (84.09%)	
No	676 (22.32%)	652 (21.53%)	1,701 (56.16%)	
Overweight				<0.001
Yes	39 (7.32%)	80 (15.01%)	414 (77.67%)	
No	640 (25.20%)	576 (22.68%)	1,324 (52.13%)	
Physical inactivity				0.005
Yes	350 (23.68%)	337 (22.80%)	791 (53.52%)	
No	329 (20.63%)	319 (20.00%)	947 (59.37%)	
Family history of stroke				0.292
Yes	56 (18.54%)	68 (22.52%)	178 (58.94%)	
No	623 (22.48%)	588 (21.22%)	1,560 (56.30%)	
Sleep duration				
Average sleep duration	(8.32 ± 0.88)	(7.75 ± 0.91)	(6.90 ± 1.20)	<0.01
Night sleep duration	(7.80 ± 0.87)	(7.34 ± 1.03)	(6.52 ± 1.09)	<0.01
Afternoon nap	(0.51 ± 0.57)	(0.40 ± 0.53)	(0.40 ± 0.53)	0.134

Table 2 presents the relationship between stroke risk and perimenopause, menopause, and sleep duration in women using multinomial logistic regression analysis. Perimenopause, menopause, average and night sleep duration were significantly associated with stroke risk (*P* < 0.01) after adjusting for age, education, marital status and employment. Furthermore, perimenopause increases the medium-risk of stroke by approximately 2–3 times (OR ranging from 3.044 to 4.200), and high-risk of stroke by about 17–30 times (OR ranging from 18.862 to 31.240). Menopause aggravates the medium-risk of stroke by approximately 3–6 times (OR ranging from 4.143 to 7.286), and high-risk of stroke by about 9–22 times (OR ranging from 10.211 to 23.970). Every 1 h increase in average sleep duration was associated with a 45.7% decrease in medium-risk (OR = 0.543; 95% CI: 0.483, 0.610), and a 67.4% decrease in high-risk (OR = 0.326; 95% CI: 0.281, 0.379). Every 1 h increase in night sleep duration was associated with a 49.3% decrease in medium-risk (OR = 0.507; 95% CI: 0.448, 0.573), and a 70.7% decrease in high-risk (OR = 0.293; 95% CI: 0.249, 0.345). It can be seen that perimenopause has a greater impact on the high-risk of stroke than menopause, while menopause has a greater medium-risk than perimenopause.

**TABLE 2:** Multivariate regression analysis on the relationships between perimenopause, menopause, sleep duration and stroke risk.

Variables	Model 1		Model 2		Model 3	
	*OR* (95% CI)	*P*	*OR* (95% CI)	*P*	*OR* (95% CI)	*P*
**Perimenopause**						
Low-risk	1.0		1.0		1.0	
Medium-risk	4.177 (2.136, 8.168)	<0.001	4.200 (2.142, 8.239)	<0.001	3.044 (1.574, 5.886)	0.001
High-risk	30.099 (5.292, 171.191)	<0.001	31.240 (5.494, 177.640)	<0.001	18.862 (3.385, 105.098)	0.001
**Menopause**						
Low-risk	1.0		1.0		1.0	
Medium-risk	7.286 (3.403, 15.602)	<0.001	7.134 (3.332, 15.276)	<0.001	4.143 (1.976, 8.685)	<0.001
High-risk	10.211 (2.021, 51.598)	<0.001	23.970 (4.570, 125.727)	<0.001	10.286 (2.037, 51.931)	0.005
Average sleep duration			–	–	–	–
Low-risk	1.0					
Medium-risk	0.543 (0.483, 0.610)	<0.001				
High-risk	0.326 (0.281, 0.379)	<0.001				
Night sleep duration	–	–			–	–
Low-risk			1.0			
Medium-risk			0.507 (0.448, 0.573)	<0.001		
High-risk			0.293 (0.249, 0.345)	<0.001		
Afternoon nap	–	–	–	–		
Low-risk					1.0	
Medium-risk					1.023 (0.829, 1.261)	0.834
High-risk					0.799 (0.605, 1.055)	0.114

Adjusted for age, education, marital status, and employment.

The relationship between sleep duration and menstrual condition (perimenopause and menopause) is shown in Table 3. Perimenopause and menopause were significantly related to average sleep duration (β = −0.483, *P* < 0.001; β = −0.841, *P* < 0.001), night sleep duration (β = −0.391, *P* < 0.001; β = −0.771, *P* < 0.001), and afternoon nap was only associated with perimenopause (β = −0.095, *P* = 0.010), suggesting perimenopause and menopausal women may sleep worse at night than pre-menopause, with no difference in naps among menopausal women.

**TABLE 3:** General linear regression on the relationships between menopause and sleep duration.

Variable	Average sleep duration		Night sleep duration		Afternoon nap	
	*B*	*P*	*B*	*P*	*B*	*P*
Age	−0.002	0.598	−0.001	0.752	−0.001	0.772
Education	0.014	0.681	0.022	0.510	−0.001	0.998
Marital status	−0.002	0.978	−0.026	0.648	0.031	0.341
Employment	−0.006	0.888	0.029	0.509	−0.028	0.253
Stroke risk	−0.658	<0.001	−0.619	<0.001	−0.024	0.196
Perimenopause	−0.483	<0.001	−0.391	<0.001	−0.095	0.060
Menopause	−0.841	<0.001	−0.771	<0.001	−0.072	0.146

**FIGURE 3:**
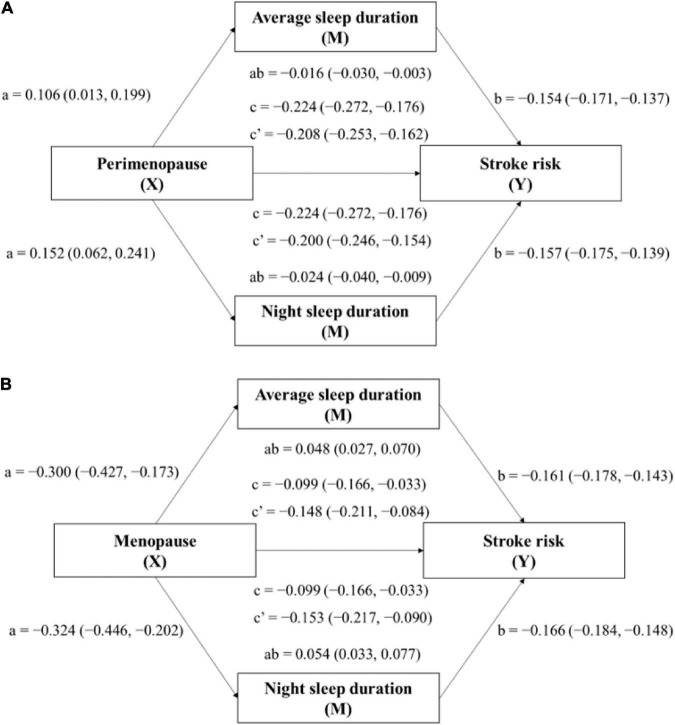
Mediation model for the relationship between menopausal status (Perimenopause: model **A**; Menopause: model **B**) and stroke risk, mediated by average sleep duration and night sleep duration. Paths a, b, c, and c′ are presented as unstandardized coefficients (95% CI). a = X on M; b = M on Y; c = total effect of X on Y; c′ = direct effect of X on Y; ab = indirect effect of X on Y. Covariates: age, education, marital status, and employment.

The mediation impact of sleep duration on the connection between menstrual status and stroke risk was found by mediating analysis, as depicted in Table 4 and Figure 3. Both perimenopause and menopause were shown to be highly associated with increased stroke risk via average sleep duration (direct effect c′ = 0.208, 95% CI: 0.162, 0.253; direct effect c′ = 0.148, 95% CI: 0.084, 0.211) and night sleep duration (direct effect c′ = 0.200, 95% CI: 0.154, 0.246; direct effect c′ = 0.153, 95% CI: 0.090, 0.217), with average sleep duration (indirect effect ab = 0.016, 95% CI: 0.003, 0.030; indirect effect ab = −0.048, 95% CI: −0.070, −0.027) and night sleep duration (indirect effect ab = 0.024, 95% CI: 0.009, 0.040; indirect effect ab = −0.054, 95% CI: −0.077, −0.033) functioning as moderators. Although there were significant correlations between both perimenopause and menopause and stroke risk after adjusting for afternoon nap (direct effect c′ = 0.225, 95% CI: 0.177, 0.273; direct effect c′ = 0.100, 95% CI: 0.033, 0.166), nap had no mediating effect (indirect effect ab = −0.001, 95% CI: −0.003, 0.001; indirect effect ab = 0.000, 95% CI: −0.001, 0.002). This suggests that night sleep duration is the key contributor to the mediating role of sleep duration between menstrual status and stroke risk, and that more emphasis should be placed on nighttime sleep than naps.

**TABLE 4:** Mediating effect of sleep duration on the relationship between menopause and stroke risk.

Independent variable	Mediating variable	Dependent variable	Coefficient (bias-corrected bootstrap 95% CI)		
Menstrual status	Sleep duration	Stroke risk	Indirect effect (ab)	Total effect (c)	Direct effect (c′)
	Average sleep				
Pre-menopause (ref.)			–	–	–
Perimenopause			0.016 (0.003, 0.030)	0.224 (0.176, 0.272)	0.208 (0.162, 0.253)
Menopause			−0.048 (−0.070, −0.027)	0.099 (0.033, 0.166)	0.148 (0.084, 0.211)
	Night sleep				
Pre-menopause (ref.)			–	–	–
Perimenopause			0.024 (0.009, 0.040)	0.224 (0.176, 0.272)	0.200 (0.154, 0.246)
Menopause			−0.054 (−0.077, −0.033)	0.099 (0.033, 0.166)	0.153 (0.090, 0.217)
	Afternoon nap				
Pre-menopause (ref.)			–	–	–
Perimenopause			−0.001 (−0.003, 0.001)	0.224 (0.176, 0.272)	0.225 (0.177, 0.273)
Menopause			0.000 (−0.001, 0.002)	0.099 (0.033, 0.166)	0.100 (0.033, 0.166)

Covariates: age, education, marital status, and employment.

**FIGURE 4:**
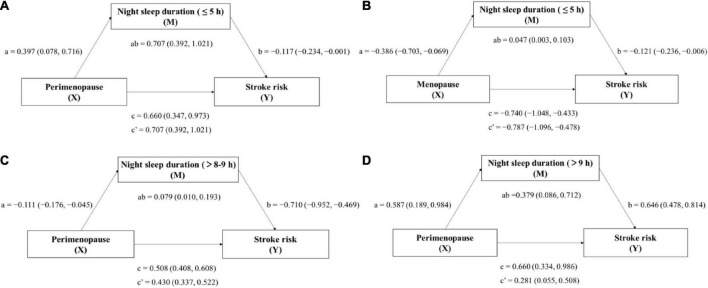
Mediation model for the relationship between menopausal status and stroke risk, mediated by night sleep duration periods (≤5 h/>8–9 h/>9 h). Model **(A)** X: Perimenopause, M: Night sleep duration(≤5 h), Y: Stroke risk; Model **(B)** X: Menopause, M: Night sleep duration(≤5 h), Y: Stroke risk; Model **(C)** X: Perimenopause, M: Night sleep duration (>8–9 h), Y: Stroke risk; and Model **(D)** X: Perimenopause, M: Night sleep duration (>9 h), Y: Stroke risk. Paths a, b, c, and c′ are presented as unstandardized coefficients (95% CI). a = X on M; b = M on Y; c = total effect of X on Y; c′ = direct effect of X on Y; ab = indirect effect of X on Y. Covariates: age, education, marital status, and employment.

The mediating effects of night sleep duration were further analyzed. Table 5 and Figure 4 present the mediating effects of duration ≤5 h per night, >5–6 h per night, >6–7 h per night, >7–8 h per night, >8–9 h per night and >9 h per night on menstrual condition and stroke risk. For night sleep duration of ≤5 h, it mediated the link between both perimenopause (indirect effect ab = 0.707, 95% CI: 0.392, 1.021) and menopause (indirect effect ab = −0.787, 95% CI: −1.096, −0.478) and stroke risk. Moreover, there was a significant association between perimenopause (direct effect c′ = 0.430, 95% CI: 0.337, 0.522) and menopause (direct effect c′ = −1.140, 95% CI: −1.268, −1.013) and stroke risk after controlling for night sleep duration of >8–9 h, while this duration only had a mediating effect (indirect effect ab = 0.079, 95% CI: 0.010, 0.193) on perimenopause and stroke risk. In terms of night sleep duration of >9 h, it also only had a mediating effect (indirect effect ab = 0.379, 95% CI: 0.086, 0.712) on perimenopause and stroke risk. Other night sleep durations did not mediate. In conclusion, only too short (≤5 h) or too long (>8–9 h/>9 h) sleep duration was found to mediate the relationship between menopausal status and stroke risk, with a positive association of perimenopause and menopause with greater stroke risk. For short night sleep duration (≤5 h), it mediated the risk of stroke both on perimenopause and menopause, whereas long night sleep duration (>8–9 h/>9 h) mediated the risk of stroke only on perimenopause. Therefore, improving women’s sleep as early as possible in perimenopause may be more effective in reducing stroke risk than after menopause.

**TABLE 5:** Mediating effect of night sleep duration on the relationship between menopausal status and stroke risk.

Independent variable	Mediating variable	Dependent variable	Coefficient (bias-corrected bootstrap 95% CI)		
Menstrual condition	Night sleep duration	Stroke risk	Indirect effect (ab)	Total effect (c)	Direct effect (c′)
	≤5 h				
Pre-menopause (ref.)			–	–	–
Perimenopause			0.707 (0.392, 1.021)	0.660 (0.347, 0.973)	0.707 (0.392, 1.021)
Menopause			0.047 (0.003, 0.103)	−0.740 (−1.048, −0.433)	−0.787 (−1.096, −0.478)
	>5–6 h				
Pre-menopause (ref.)					
Perimenopause			0.010 (−0.002, 0.022)	0.030 (−0.109, 0.169)	0.021 (−0.114, 0.155)
Menopause			0.009 (−0.012, 0.027)	0.237 (0.083, 0.391)	0.228 (0.080, 0.377)
	>6–7 h				
Pre-menopause (ref.)					
Perimenopause			−0.013 (−0.027, −0.003)	0.040 (−0.026, 0.106)	0.053 (−0.012, 0.119)
Menopause			0.010 (−0.002, 0.025)	0.130 (0.047, 0.213)	0.121 (0.038, 0.203)
	>7–8 h				
Pre-menopause (ref.)					
Perimenopause			0.191 (0.152, 0.229)	0.174 (0.114, 0.235)	−0.016 (−0.048, 0.015)
Menopause			−0.150 (−0.234, −0.068)	−0.014 (−0.113, 0.085)	0.136 (0.087, 0.185)
	>8–9 h				
Pre-menopause (ref.)					
Perimenopause			0.079 (0.010, 0.193)	0.508 (0.408, 0.608)	0.430 (0.337, 0.522)
Menopause			0.008 (−0.030, 0.059)	−1.132 (−1.234, −1.030)	−1.140 (−1.268, −1.013)
	>9 h				
Pre-menopause (ref.)					
Perimenopause			0.379 (0.086, 0.712)	0.660 (0.334, 0.986)	0.281 (0.055, 0.508)
Menopause			−1.202 (−2.021, −0.713)	−1.383 (−2.112, −0.654)	−0.181 (−0.772, 0.410)

Covariates: age, education, marital status, and employment.

## Discussion

Perimenopause and menopause were shown to be strongly linked to an increased risk of stroke in this research, night sleep duration acting as the key contributor to average sleep duration as the mediating factor. Further investigation into the impact of nighttime sleep duration periods revealed that only too short (≤5 h) and long (>8–9 h/>9 h) night sleep duration partially mediated the association between perimenopause and menopause and stroke risk. This was the first study that we were aware of that explored the effect of sleep duration in mediating the relationship between menopausal status and stroke risk in Chinese women.

Our findings also confirmed that the onset of menopause was associated with the increased risk of stroke, which was consistent with previous studies. According to a brief review in the United States, women experiencing menopause had a higher risk of stroke than males (Shekhar et al., 2017). An experimental study suggests that perimenopause prompts an innate immune inflammatory response in the female reproductive organs to spread to the brain, making the brain more susceptible to ischemic damage (Raval et al., 2019). This is most likely due to postmenopausal women’s lower levels of sex hormones such as estrogen and progesterone. These alterations disrupt estrogen receptor-dependent signaling pathways, increasing the risk of stroke and its severity (Bustamante-Barrientos et al., 2021).

A Canadian longitudinal study on aging showed that perimenopausal and postmenopausal women have significant rates of sleep disruption and insomnia complaints (Zolfaghari et al., 2020), which confirmed our conclusion. Up to 82% of women going through menopause have vasomotor symptoms such as hot flushes and night sweats (Kapral and Bushnell, 2021), which may lead to sleep disorders during perimenopause and menopause, as mentioned in the systematic review (Muka et al., 2016a). One possible explanation for these findings is that perimenopausal and postmenopausal women are vulnerable to many factors affecting their sleep, and the majority of them are sleep-deprived, so prolonged sleep duration could help reduce the risk of stroke. Our findings also matched the result of the China Health and Retirement Longitudinal Study (Fang and Zhou, 2019), which found no statistical significance between menopausal state and daytime napping. Adequate night sleep duration tended to present a lower incidence of stroke risk for menopausal women.

Several epidemiological studies (Fang et al., 2014; Akinseye et al., 2016; Wang et al., 2022) have explored the relation between sleep duration and stroke risk, but the results have been inconsistent due to discrepancies in adjustments, sample sizes, research methods, and study end points. Average sleep duration and night sleep duration were both shown to be inversely related with stroke risk in our research, whereas the afternoon nap was not found to be favorably associated with stroke risk. A review also suggested that sleep deprivation screening should be regarded as part of community-based primary stroke prevention, and the significance of adequate sleep needs to be highlighted (Guzik and Bushnell, 2017), which was consistent with our findings. Results from the China Health and Retirement Longitudinal Study also showed that taking naps was not linked to a higher risk of stroke, which in line with our conclusion (Baker et al., 2018). However, a meta-analysis of pooled data from cohort studies found no causal relationship between sleep duration and stroke risk (Banach et al., 2020), and a dose-response meta-analysis of prospective cohort studies found a slightly lower risk of ischemic stroke among short duration sleepers (He et al., 2017), both of which were not supported by our findings.

Sufficient, restorative sleep is essential for the proper operation of all bodily systems. Many sleep issues in women occur with menopause and might be connected with vasomotor symptoms due to changes in the postmenopausal profile of sex hormones (e.g., hot flashes and night sweats) (Baker et al., 2018). Estrogen deficiency in particular has been postulated to contribute to the sleep difficulties that women often begin to experience in their perimenopausal period, and then increasingly with the failure of ovarian. Aging, along with narrowing of the upper respiratory tract, produces snoring may predict cardiovascular and cerebrovascular diseases and overall rates of death. Most elderly women suffer from sleep disorders on the one hand and cardiovascular and cerebrovascular risks caused by snoring on the other. Poor sleep quality and excessive sleep duration are linked to worse stroke outcomes (Beverly et al., 2020). The average sleep duration was discovered to have a partial mediating effect on the association between menopausal status and stroke risk, and night sleep duration acted as the key contributor. A possible physiological justification is that rapid eye movement (REM) sleep, which involves brain recovery in sleep circles, because REM is proportionately connected to sleep duration (Pace et al., 2018). Nighttime sleep contributes considerably to average sleep duration, while quick naps may not produce an effective REM due to their normal short duration. Meanwhile, abnormal hormone levels due to ovarian failure affect the sleep-wake cycle, leading to a reduction in the number and duration of REM, which contributes to sleep disorders. A practical implication of this finding is that the stroke risk may be partly counteracted by acquiring sufficient night sleep. Therefore, we should pay more attention to the duration and quality of our nighttime sleep. We may be able to use electroencephalograph (EEG) to monitor women’s pattern of sleep waveform, and use appropriate clinical interventions to maintain the normal number and duration of REM, and REM is not affected by abnormal hormone levels, so as to achieve maintenance of high sleep quality and reduce the risk of cardiovascular and cerebrovascular diseases. What’s more, when perimenopausal and postmenopausal females were separated by night sleep duration categories for further analysis, surprisingly, only short night sleep duration mitigate the association between menopause and stroke risk, but a broader range of sleep duration periods (both short and long) could mediate the relationship between perimenopause and stroke risk. This means that intervention or prevention of sleep problems in women during perimenopause may be more effective in reducing stroke risk than during menopause.

Since the effect sizes of sleep were very modest, other potential sleep-related mediating variables (e.g., sleep quality, disruptions, and variability) need to be examined in future research to provide a more thorough knowledge of mechanisms responsible for the relationship between menopause and stroke risk.

There are several limitations to this study as well. First, self-reported questionnaires were employed to obtain data on average sleep duration and menopause rather than objective measurements. Although self-reported sleep duration has been one of the most commonly utilized approach, sleep duration recorded by actigraphy-measured assessments should be more trustworthy, yet objective sleep duration data in large population research is impractical to collect. Second, concomitant sleep problems such as snoring, nocturnal waking, apnea, and somnolence, and the use of medications, such as contraception pills, hormone therapy, steroids medication for any inflammatory condition, or antidepressants, would have an effect on sleep duration to some extent. These should be included as variables in future research. Third, as with all cross-sectional research, we were unable to confirm causality; nevertheless, mediation analysis might be used to evaluate alternative pathways based on association.

## Conclusion

Our research found a significant link between perimenopause, menopause and stroke risk among Chinese suburban women. The night sleep duration was mainly found to mediate the link between menopause and the risk of stroke. The risk of stroke in perimenopausal women was more broadly influenced by night sleep duration than in menopause. This finding revealed that the night sleep duration could be a potential mechanism between menopause and stroke risk, and that early sleep interventions during perimenopause may be effective in improving stroke risk for women.

## Data availability statement

The raw data supporting the conclusions of this article will be made available by the authors, without undue reservation.

## Ethics statement

The studies involving human participants were reviewed and approved by Ethics Review Committee of the Shanghai University of Medicine and Health Sciences (No. 2019-SMHC-01-003). The patients/participants provided their written informed consent to participate in this study.

## Author contributions

PZ and TW: conceptualization. CD: data curation. MP and SP: investigation. XL and JZ: writing—original draft. PZ: writing—review and editing. All authors have read and agreed to the published version of the manuscript.
